# A Non-Destructive Distinctive Method for Discrimination of Automobile Lubricant Variety by Visible and Short-Wave Infrared Spectroscopy

**DOI:** 10.3390/s120303498

**Published:** 2012-03-12

**Authors:** Lulu Jiang, Fei Liu, Yong He

**Affiliations:** 1 Zhejiang Technology Institute of Economy, Hangzhou 310018, China; E-Mail: abbyu111@sina.com; 2 College of Biosystems Engineering and Food Science, Zhejiang University, Hangzhou 310058, China

**Keywords:** lubricant, visual and short-wave spectroscopy, wavelet packet transform, uninformative variable elimination, simulated annealing algorithm

## Abstract

A novel method which is a combination of wavelet packet transform (WPT), uninformative variable elimination by partial least squares (UVE-PLS) and simulated annealing (SA) to extract best variance information among different varieties of lubricants is presented. A total of 180 samples (60 for each variety) were characterized on the basis of visible and short-wave infrared spectroscopy (VIS-SWNIR), and 90 samples (30 for each variety) were randomly selected for the calibration set, whereas, the remaining 90 samples (30 for each variety) were used for the validation set. The spectral data was split into different frequency bands by WPT, and different frequency bands were obtained. SA was employed to look for the best variance band (BVB) among different varieties of lubricants. In order to improve prediction precision further, BVB was processed by UVE-PLS and the optimal cutoff threshold of UVE was found by SA. Finally, five variables were mined, and were set as inputs for a least square-support vector machine (LS-SVM) to build the recognition model. An optimal model with a correlation coefficient (*R*) of 0.9850 and root mean square error of prediction (RMSEP) of 0.0827 was obtained. The overall results indicated that the method of combining WPT, UVE-PLS and SA was a powerful way to select diagnostic information for discrimination among different varieties of lubricating oil, furthermore, a more parsimonious and efficient LS-SVM model could be obtained.

## 1. Introduction

Automobile lubricating oil is a kind of efficient anti-friction agent, mainly used to reduce the friction between the surfaces of moving parts. At present, there are many different varieties of lubricating oil, and these varieties greatly affect the market price and quality. Recently, consumption of lubricating oil has increased, as a result, instances of fraud have increased as well. To make enormous profits, some illegal factories mix varieties of lubricating oil of different quality, and such behaviors infringe on the rights and interests of consumers and legal factories. Therefore, there is a need to develop an accurate and rapid method to discriminate between varieties and qualities of lubricants, which may be also utilized for the detection of adulteration.

Recently, some researchers have devoted much attention to the study of lubricants. Zhao *et al*. studied the content of wear metal in lubrication oil by ICP-AES [1]. Wei *et al*. analyzed the additive element contents in lube oil by atomic emission spectra [2]. Borin *et al*. applied mid-infrared spectroscopy for quantification of contaminants in lubricating oil [3]. However, there are few reports on the discrimination of different varieties of lubricants using VIS-SWNIR. With the development of modern chemometrics and instrumentation, VIS-SWNIR has proved to be useful in providing a cheap and rapid detection technology [4–7], which is widely applied to discriminate agricultural and food production, such as yogurts [4], fruit vinegars [5], Chinese bayberries [6] and tea plants [7]. However, the spectra typically consist of broad, weak, non-specific and overlapping bands [8], and the data matrix of the spectra is often very large, so how to obtain the most useful information accurately from the data matrix is of great importance.

Wavelet packet transform (WTP), an extension of wavelet transform (WT) [9], is a powerful signal processing technique. It transforms the raw spectral data into different frequency bands, and the frequency component in different bands has different contribution to the multivariate model [10], so how to find the most useful band that represents the most variant spectral information is an important issue.

Uninformative variable elimination by PLS (UVE-PLS) is a method for variable selection [11]. The method evaluates the reliability of each variable in the model based on analysis of regression coefficients of PLS and selection criterion. It has been widely applied in analytical chemistry for removing the low-frequency varying background and the high-frequency noise [10], retention prediction of peptides [12], and analysis of steroids [13]. In these researches, they proposed to use an artificial random variable matrix with very small amplitude, added to the original data set to estimate the cutoff. This method is experiential.

The simulated annealing (SA) algorithm, a simulation of the annealing process used for metals, was put forward by Kerkpatrick in 1983 [14]. It offers arguably the simplest and the most elegant solution with the “best” record for solving combinatorial optimization problems. Unlike other algorithms, the SA algorithm allows various types of transitions in which some of them may be opposite the goal [15]. Hence, the SA algorithm has been widely applied to many optimization problems, such as multi-objective optimization of a constrained problem [16], the maximum clique problem [17] and multiparameter analysis of water optical properties from above-water remote-sensing reflectance [18]. However, the application of WPT combined with SA in extraction of variance spectra has received little attention with no contributions reported in the literature.

In this work, WPT with SA was used to search for BVB, then irrelevant variables in BVB were eliminated by UVE-PLS, and SA was employed to search for the cutoff threshold of UVE-PLS. Finally, the variables were mined as input sets for a LS-SVM to build a lubricant recognition model.

## 2. Experimental Section

### 2.1. Sample Preparation

A total of 180 lubricant samples were used as the whole data set. The calibration set of 90 samples was selected randomly for the optimal parameters. The remaining 90 samples were selected as validation set to evaluate the performance of discrimination model. The 180 samples were purchased in local market including the following three varieties: Changcheng (Cc), Huaxiayyangguang (Hxyg), Caltex (Ca). All samples were stored in the lab at a constant temperature of 25 ± 1 °C to equalize the temperature.

### 2.2. Spectral Measurement

For each sample, three reflectance spectra were scanned by a handheld FieldSpec Pro FR (325–1,075 nm)/A110070 (Analytical Spectral Devices Inc., Boulder, CO, USA). The light source consists of a Lowell pro-lam interior light source assemble/128930 with a Lowell pro-lam 14.5 V Bulb/128690 tungsten halogen bulb that could be used both in the visible and near infrared regions. The field-of-view of the spectral radiometer is 10°. The spectroradiometer was placed at a height of approximately 250 mm and at a 45° angle away from the center of the sample. The light source was placed at a height of approximately 150 mm and 45° angle away from the sample. The spectrum of each sample was the average of 30 successive scans with 1.5 nm intervals. Three spectra were collected for each sample and the average spectrum of these two measurements was used in the later analysis. All spectral data were stored in a computer and processed using the RS3 software for Windows (Analytical Spectral Devices) designed with a Graphical User Interface.

### 2.3. Algorithm

#### 2.3.1. Wavelet Packet Transform

**Figure 1.**
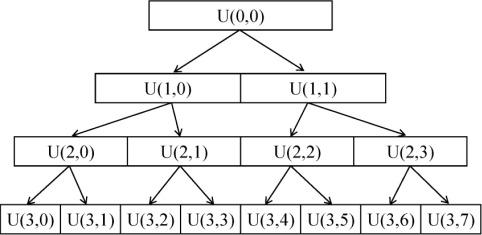
Full WPT binary tree.

WPT is a derivative of WT. In the fast wavelet transform (WT) [19], a partial multi-resolution analysis is performed. Only approximation coefficients (low-pass node) are employed to deduce both scale and wavelet coefficients at the next resolution level. However, WPT allows a full multi-resolution analysis; both the approximation and detailed coefficients (high-pass node) are involved to decompose at the next resolution level at the same time [20]. As a result, a library of sub-bands including low frequency and high frequency is obtained. A schematic diagram for the WPT decomposition was shown in Figure 1. In the diagram, each node is identified by the pair of indices *U* (*j*,*k*), where *j* is the level of decomposition and *k* is the position of the node at that level of decomposition.

#### 2.3.2. Uninformative Variable Elimination by Partial Least Squares (UVE-PLS)

In linear least squares models, the predictions *ŷ* are computed by the equation:

(1) $$\hat{y}=\mathrm{Xb}+e$$

where *X* is a *n* × *p* matrix containing p spectral responses of *n* samples, *b*(1, *p*) is the vector of PLS regression coefficients and *e*(*n*, 1) is vector of errors that cannot be explained by the model.

In UVE-PLS method, a PLS regression coefficient matrix *b* = [*b_1_*,…*b_n_*] is calculated through a leave-one-out validation [8,11], then the reliability of each variable (wavelength) can be quantitatively measured by its stability; the stability of variable *j* can be calculated as:

(2) $$S_{j}=\mathrm{mean}(\beta_{j})/\mathrm{std}(\beta_{j})$$

where *mean*(*β_i_*) and *std*(*β_i_*) are the mean and standard deviation of the regression coefficients of variable *j*, so, the larger the stability, the more important the corresponding variables is, and the variables whose stability is lower than a cutoff threshold are regarded as uninformative and should be eliminated.

#### 2.3.3. Principle of SA Algorithm

In simulated annealing, a problem starts with an initial solution, and this solution can be easily changed. But as the temperature *t* is decreased, changing the configuration is increasingly difficult. Finally, if *t* is lowered sufficiently, no further changes in the solution space are possible. To avoid being frozen at a local optimum, the SA algorithm moves slowly through the solution space. This controlled improvement of the objective value is accomplished by accepting non-improving moves with a certain probability that decreases as the algorithm progresses. This criterion is a Boltzman’s probability distribution (Metropolis criterion) as a function of temperature *t*:

(3) $$p(\Delta d)=\text{exp}\left(\frac{-\Delta d}{t}\right)$$

where

(4) $$\Delta d=d(x^{\prime })-d(x_{i})$$

*d* is a distance operator, and *x′* and *x_i_* are current values.

The main processes of the SA algorithm for optimal parameters are explained as follows:

Step 1: Initialize parameters: set initial temperature *T_I_*, termination temperature *T_s_*, cooling coefficient *τ* of temperature, and select an initial solution *x_1_* in solution space *X*. Finally, input the best value *F^best^* and the corresponding solution:
  $$x^{\mathrm{best}}$$
  $$x^{\mathrm{best}}=x_{1}$$
  $$F^{\mathrm{best}}=F(x_{1})$$
  $$T=T_{I}$$
Step 2: Another solution *x_2_* is generated at random in the neighbor to *x^best^*.
  If *F*(*x*_2_) < *F^best^* then *F^best^ ← F*(*x*_2_) and *x^best^* ← *x*_2_
  If *F*(*x*_2_) > *F^best^* then, consider *R* (a random number in [0, 1]):
    $$\Delta =F(x_{2})-F^{\mathrm{best}}$$
    $$p=\frac{1}{1+\text{exp}(\frac{-\Delta d}{T})}\sigma^{2}$$
  If *R* < *P* then *x^best^* ← *x*_2_
  Else change = false
Step 3: Check whether the stopping criteria are satisfied. If satisfied, end the SA algorithm; otherwise, change annealing temperature:
  T=T*τ
  and back to step 2.

## 3. Results and Discussion

### 3.1. Absorbance Spectra of Three Varieties of Lubricants

**Figure 2.**
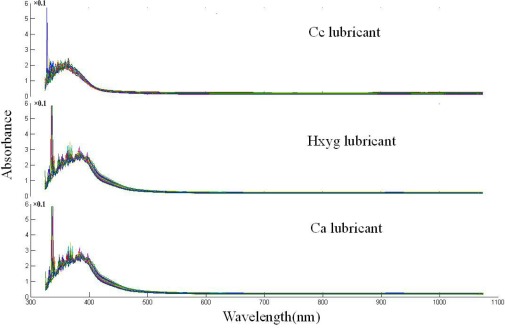
Vis-SWNIR spectra of three varieties of lubricant.

Typical spectra of three varieties of lubricants are shown in Figure 2. Due to potential system imperfection, the scattering ray affects the accuracy of measurement; there is lots of noise in the 325–400 nm region. The spectra of samples from all three varieties have similar gross patterns of absorbance, there is a sharp absorption peak round 380 nm. After 400 nm, the absorbance values begin to decrease, and the spectral curve is flat. Though the trend of the spectra is similar, some latent differences and features exist according to the chemical components and color variance which could not be distinguished by the naked eye. With a closed observation, a small difference between Cc lubricant and the two other lubricants existed in the 400–450 nm region, which might have resulted from the color variance.

### 3.2. WPT Decomposition

Before SA was employed to search for BVB, the searching range of SA should be defined; the range includes lower and upper bound constraints of decomposition level; the max node number in each level. Lower and upper bound constraints of WPT decomposition should be determined first, upper bound, namely, for a signal with a length of *N*, the theoretically maximum decomposition level *J*, which could be calculated by the Matlab command of “wmaxlev”. wmaxlev is a one- or two-dimensional wavelet or wavelet packets-oriented function. wmaxlev can help avoid unreasonable maximum level values. wmaxlev gives the maximum allowed level decomposition, but in general, a smaller value is taken. After some performance comparison, the decomposition scale could be determined. The lower bound of decomposition was defined as zero, namely, the signal has not been decomposed. Then, constraint range of node number was determined according to decomposition level, because the WTP decomposed signal is the full binary tree (see Figure 1), the upper bound of node number was set as *2^l^*, where *l* is the decomposition level. The low bound number of the node was zero. In this work, the wavelet functions within the range of *Db1*–*Db9* were computed for a better performance, and the *Db4* wavelets function was employed as the optimal one after performance comparison and all the calculations were performed using MATLAB 7.6 (Mathworks, Natick, MA, USA).

### 3.3. SA Configuration Variables

#### 3.3.1. Determination of SA Algorithm Parameters

After different bands were obtained by WPT decomposition, SA was employed to seek this optimal band in this study. Before using of SA, some parameters of SA algorithm should be preset:

1. Selection of initial temperature *T_I_*, and termination temperature *T_s_*: Initial temperature *T_I_* is 100 °C, and termination temperature *T_s_* is 0 °C.
2. State generate function and state accepting function: Student’s *t* distribution was employed to generate new solutions in the SA algorithm. The random disturbance can be regarded as a jumping optimal model. State accepting function uses the change in function values between the current point and the new point to determine whether the new point is accepted or not.
3. Annealing schedule: Annealing schedule is an exponential annealing schedule which updates the current temperature based on the initial temperature and the current annealing parameter *k* (the number of evaluations of the objective function):
  (5) $$T_{n+1}=0.95^{K}T_{n}$$
4. Algorithm termination criteria: In general, there are two stopping rules: one is that the number of temperature transitions satisfies the temperature termination rules, and the other one is that the neighbor solution was not improved after a certain period [21]. In our strategy, the algorithm stops when the average change on the value of the fitness function at current point is less than 1e–6 after 500 iterations.

#### 3.3.2. Evaluation of Fitness Function

The performance of SA was evaluated through a fitness function, also known as objective function. The function value was the criterion for guiding SA to the global optimum. The prediction ability of the calibration model was evaluated with parameters of correlation coefficient (*R*) and root mean and root mean square error of calibration (RMSEC) or prediction (RMSEP), and the ideal model should have high *R* value, and low RMSEC and REMSEP values [22]. So the fitness function could be defined as follows:

(6) $$\text{max}\;f(X)=\frac{R}{1+\mathrm{RMSEC}}=\frac{\sum_{i-1}^{n}\left(c_{i}^{p}-{\bar{c}}^{p})(c_{i}^{0}-{\bar{c}}^{0}\right)}{\left(1+{\left(\frac{1}{n}\sum_{i-1}^{n}{\left(c_{i}^{0}-c_{i}^{p}\right)}^{2}\right)}^{\frac{1}{2}}\right)\cdot {\left(\sum_{i-1}^{n}{\left(c_{i}^{p}-{\bar{c}}^{p}\right)}^{2}\cdot \sum_{i-1}^{n}{\left(c_{i}^{0}-{\bar{c}}^{0}\right)}^{2}\right)}^{\frac{1}{2}}}$$

where $c_{i}^{0}$ is the actual concentration of spectrum *i* in the calibration set, *c̄*^0^ is the mean of $c_{i}^{0}$, $c_{i}^{p}$ is the concentration predicted by the model, *c̄^p^* is the mean of $c_{i}^{p}$, and n is the number of calibration set. The fitness function was measured in the form of leave-one-out cross-validation using partial least-square regression (PLSR), principal components (PCs) of PLSR according to accumulative reliabilities of PCs [23]. Thus the model was built using *n*−1 training samples and the one left out is used for prediction.

### 3.4. Optimization Results

**Figure 3.**
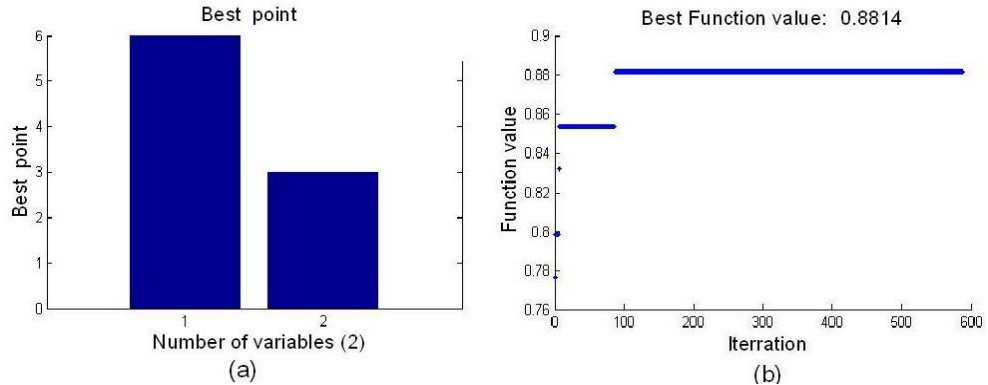
Result of optimal node by SA. (**a**) Optimal node. (**b**) Best fitness function value.

After the parameters of WTP and SA were determined, SA was employed to search for the BVB. The optimal BVB and the best function value are shown in Figure 3, and the optimal node was (6,3). The best fitness function value of 0.8814 was obtained by SA.

**Figure 4.**
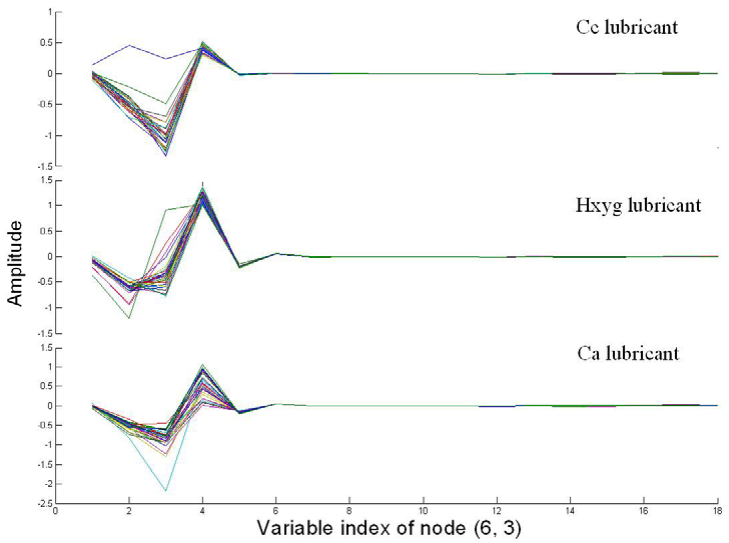
Characteristics of coefficients of node (6,3).

Figure 4 shows the coefficients of node (6,3) of three varieties of lubricants. Compared with Figure 2, there was obviously variance among three varieties of lubricants, which could be discriminated by the naked eye. Moreover, it also could be found from Figure 4, that the trend of the curve of the same variety was very similar, except there was only an abnormal curve in the Cc lubricant.

### 3.5. Development and Performance of LV-SVM

Support vector machine (SVM) is a state-of-the-art statistical learning method proposed by Vapnik [24], which has a good theoretical foundation in statistical learning theory based on the principal of structural risk minimum (SRM). LS-SVM can be considered as an extension of SVM [25], which is a new and attractive statistical learning method, and has the capability of dealing with linear and nonlinear multivariate calibration. When using LS-SVM, three crucial problems are required to be solved, including the determination of the optimal input feature subset, proper kernel function, and the optimal kernel parameters [26]. Currently, there is no systematic methodology for selection of kernel functions. In our work, based on experience, a radial basis function (RBF) was adopted as the kernel function of LS-SVM, the regularization parameter gamma (*γ*) within the region of (2^−1^, 2) and the parameter *sig2* (*σ*^2^) within the region of (10^−2^, 10^4^) were set.

**Figure 5.**
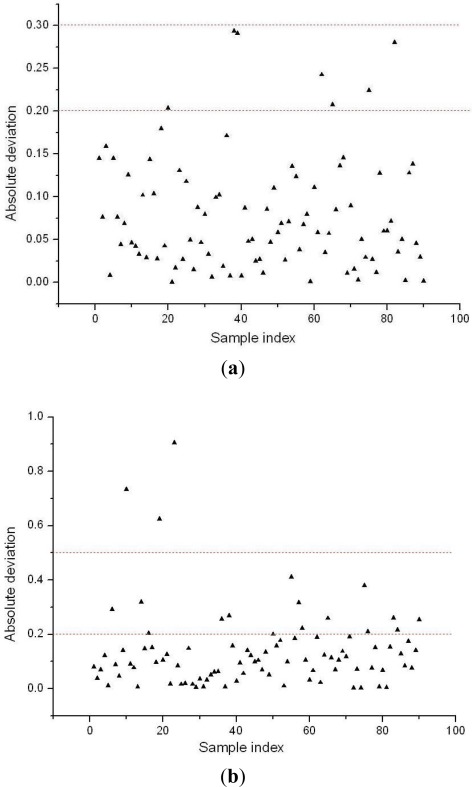
The absolute deviation value of prediction results of validation set (sample index 1–30 for Cc lubricant, 31–60 for Hxyg lubricant, and 61–90 for Ca lubricant). (**a**) BVB as the input set for LS-SVM. (**b**) Full spectra as the input set for LS-SVM.

Finally, BVB of 18 variables were extracted, and they constituted a new set of variables which could be used as inputs for LS-SVM (BVB-LS-SVM) to build the recognition model. In the application of BVB-LS-SVM, each variety of lubricant in the calibration set was assigned a dummy variable as a reference value (set Cc lubricant as 1, set Hxyg lubricant as 2, set Ca lubricant as 3). A total of 90 samples in the validation set were predicted by the LS-SVM model. Good performance was achieved, and the prediction results for *R* and RMSEP were 0.9920 and 0.1042. The absolute deviation of prediction results of each sample is shown in Figure 5(a). The threshold error of recognition was set as ±0.1 (the red dotted lines in the Figure 5). Taking Cc lubricant for example, the standard value for Cc is 1, and if the predicted value is lower than 0.9 or higher than 1.1, it will be marked as a wrong identification, so when the threshold error was ±0.3, the total discrimination rate was 100%, if the threshold error was set as ±0.2, the prediction results indicated only seven samples (No. 20 Cc, No. 38, 39, Hxyg, and No. 62, 65, 75, 82, Ca) were wrongly misclassified. Figure 5(b) shown the absolute deviation of prediction results of each sample when the full spectral variables were used as inputs for LS-SVM. When threshold error was set as ±0.2, the prediction rate was only 80%, even when the threshold error was set as ±0.5, three samples still exceeded this limit. Therefore, The BVB-LS-SVM model not only significantly improved prediction accuracy, but also compressed the raw spectral data greatly.

### 3.6. Elimination of Uninformative Variables in BVB

In order to improve prediction accuracy further, uninformative variable elimination by PLS (UVE-PLS) was employed to remove uninformative variables of BVB. In the UVE-PLS method, how to estimate the cutoff is a very important issue. In the previous research, they used artificial random variables, added to the data set to calculate the cutoff [11], and this way was experiential. In order to overcome this defect, in this work, SA was employed to search for the optimal cutoff of UVE-PLS; the fitness function of SA was the same as Equation (6). The number of principal components (PCs) for PLS was also according to the accumulative reliabilities of PCs, here, the number of PCs was six.

**Figure 6.**
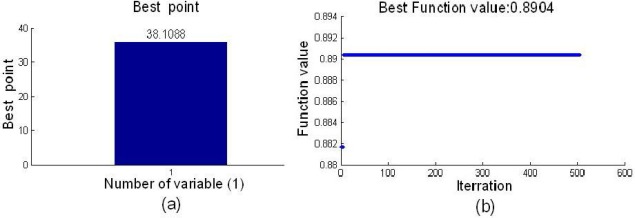
Result of optimal cutoff by SA. (**a**) Optimal cutoff. (**b**) Best function value.

**Figure 7.**
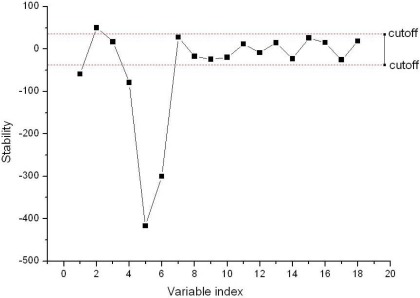
Stability distribution of each variable, and the two red dotted lines indicate the lower and upper cutoff.

Figure 6 shows the result of the optimal cutoff and the best fitness function, and the optimal cutoff was 38.1088, and the best fitness function value of 0.8904 was obtained by SA. The stability of each variable in the BVB was shown in Figure 7. In the Figure 7, the dotted lines show the cutoff threshold, the vertical bar in Figure 7 indicated the stability range. Variables whose stability lies within the dotted lines were eliminated, and the variables whose stability lies out of the dotted lines were retained. Finally, only five variables in the BVB were retained.

**Figure 8.**
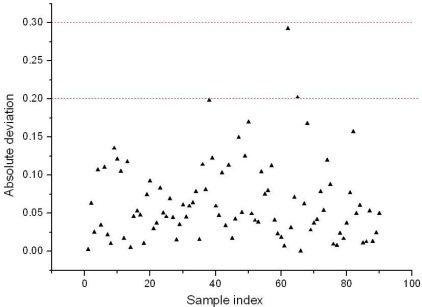
The absolute deviation value of prediction results of BVB-RV-LS-SVM.

The retained five variables constituted a new set of variables for LS-SVM (BVB-RV-LS-SVM). The performance of BVB-RV-LS-SVM was evaluated by the same 90 unknown samples mentioned above, and *R* of 0.9950 and RMSEP of 0.0829 were obtained, respectively. The absolute deviation of prediction results of each sample is shown in Figure 8. As one could see from Figure 8, when the threshold error was set as ±0.3, no sample was mistakenly discriminated, if the threshold error was ±0.2, there were only two misclassified samples, these were samples 62 and 65, and the total discrimination rate reached 97.78%. Therefore, compared with BVB-LS-SVM, the BVB-RV-LS-SVM model not only improved the prediction accuracy greatly, but also produced a more parsimonious model.

### 3.7. Comparison of Different Calibration Models

In this section, the method proposed in this work was compared with the conventional methods:

Method I: LS-SVM model with certain LVs as the inputs [5]. Partial least squares discriminant analysis (PLS-DA) was also applied as a way to extract the Latent variables (LVs) which were determined by the lowest value of predicted residual error sum of squares (PRESS). Then, selected LVs were employed as the input sets of LS-SVM according to their explained variance.
Method II: Partial least squares regression (PLSR) [23].
Method III: LS-SVM with optimal wavelengths. SA was employed to search for the optimal wavelength in the full spectrum; this method is equivalent to a genetic algorithm (GA) searching for the optimal wavelength from the full spectrum.
Method IV: LS-SVM with certain wavelengths. Certain wavelengths were selected from the full spectrum by UVE-PLS, and optimal cutoff threshold of UVE-PLS was searched by SA.
Method V: The method proposed in this work (BVB-RV-LS-SVM).

Compared with all the models above, the model presented in this work achieved satisfactory prediction results (seen in Table 1). It was clear that using BVB-RV-LS-SVM, with only five variables, the prediction result could be slightly better than that of method IV using 151 variables (wavelengths). For the calibration set, the highest correlation coefficient *R* of 0.9943 and lowest RMSEC of 0.0878 were obtained by BVB-RV-LS-SVM. This result demonstrated that good best performance was achieved; the results also indicated that these five mined variables might be the best diagnostic information for the discrimination of varieties of lubricants. Furthermore, *R* for calibration and validation set, RMSEP and RESEC were very close, which indicated that almost no overfitting happened, and a good stability and generalization was achieved by this new model. Therefore, according to the result of calibration and validation set, the five variables as feature information could represent the main variance information, and these variables could be applied instead of the whole original spectra to discriminate the varieties of lubricants. Moreover, these mined variables could be used as diagnostic information for the development of lubricant variety discrimination apparatus.

## 4. Conclusions

The variety discrimination of lubricants was successfully performed by Vis-SWNIR spectroscopy with a hybrid method combination of WPT, UVE-PLS, SA and LS-SVM. Using the WPT, UVE_PLS and SA, the raw spectra data set was greatly compressed, and only five variables were mined. Then, these variables were used as input set for LS-SVM to build a recognition model, and good performance was achieved. The overall results indicated that the method combining WPT, UVE-PLS and SA was a powerful way of compressing the spectral data set and selection of diagnostic information.

## Figures and Tables

**Table 1.** The discrimination results of calibration and validation sets by different calibration models.

**Parameters**	**Method I**	**Method II**	**Method III**	**Method IV**	**Method V**
PCs/LVs/Sw/Sv ^a^	6	6	389	151	5
Calibration Set					
*R*^b^	0.9845	0.9539	0.9864	0.9844	0.9943
RMSEC^c^	0.1433	0.2540	0.1351	0.1472	0.0878
Validation Set					
*R*^b^	0.9511	0.9256	0.9844	0.9950	0.9950
RMSEP ^c^	0.2718	0.3194	0.1472	0.0829	0.0827

a PCs/LVs/Sw/Sv: Principle components/latent variables/Selected wavelengths/Selected variables.

b *R*: correlation coefficient.

c RMSEC/RMSEP: root mean square error of calibration or prediction.
